# 

**Published:** 1892-10

**Authors:** 


### Society Meetings.

The American Electro-Therapeutic Association will hold its second annual meeting in New York, at the Academy of Medicine, 17 West Forty-third street, October 4, 5, and 6, 1892.

Papers are announced by Drs. George J. Engelmann, Wellington Adams, and George F. Hulbert, of St. Louis; William F. Hutchinson, of Providence, R. I.; Franklin H. Martin, of Chicago, Ill.; A. Lapthorn Smith, of Montreal, Canada; R. J. Nunn, of Savannah, Ga.; Thomas W. Poole, of Lindsay, Ont.; C. Eugene Riggs, of St. Paul; W. J. Herdman, of Ann Arbor, Mich.; D. S. Campbell, of Detroit, Mich.; G. Betton Massey, of Philadelphia; Henry D. Fry, of Washington, D. C.; H. E. Hayd, of Buffalo, N. Y.; J. H. Kellogg, of Battle Creek, Mich.; C. G. Cannaday, of Roanoke, Va.; Ernest Wende, of Buffalo, N. Y.; and William J. Morton, Augustin H. Goelet, A. D. Rockwell, Landon Carter Gray, Robert Newman, Ephraim Cutter, Frederick Peterson, G. M. Hammond, F. Van Raitz, of New York, and many others. Dr. J. Mount Bleyer will give an instructive lecture, with demonstrations, entitled, The Phonograph and Microphonograph, the Principles Underlying Them and Their Uses in the Sciences.

In connection with the meeting, there will be an exhibition of modern medical electrical apparatus, all the prominent manufacturers being represented.

The Southern Surgical and Gynecological Association appointed its fifth annual meeting to be held in Louisville, November 8th. This is found to fall on the day of the presidential election, hence the Council has wisely changed the date, and the meeting will be held Tuesday, Wednesday and Thursday, Nov. 15, 16, and 17, 1892, at Louisville, Ky. Dr. J. McFadden Gaston, of Atlanta, is president, and Dr. L. S. McMurtry, of Louisville, is chairman of the committee of arrangements. The secretary, Dr. W. E. B. Davis, of Birmingham, is planning an elaborate programme for the meeting.

---

The New York State Association of Railway Surgeons will hold its second annual meeting at the Academy of Medicine, 17 West 43d street, New York, on November 14, 1892. The profession is cordially invited.

### BOOKS RECEIVED.

A Text-book of Morbid Histology for Students and Practitioners. By Rubert Boyce, M. B., M. R. C. S., Assistant Professor of Pathology in University College, London. With 130 colored illustrations. New York: D. Appleton & Co., 1, 3, and 5 Bond street. 1892.

A Practical Treatise on Diseases of the Skin. By John V. Shoemaker, A. M., M. D. Professor of Skin and Venereal Diseases in the Medico-Chirurgical College and Hospital of Philadelphia. Physician to the Philadelphia Hospital for Diseases of the Skin; Member of the American Medical Association, of the Pennsylvania and Minnesota State Medical Societies, of the American Academy of Medicine, and of the British Medical Association; Fellow of the Medical Society of London. Second edition, revised and enlarged, with chromogravure plates and other illustrations. New York: D. Appleton & Co. 1892. Price, $5.00.

Geographical Pathology: An Inquiry into the Geographical Distribution of the Infective and Climatic Diseases. In two volumes, by Andrew Davidson, M. D., F. R. C. P., Ed., Late Visiting and Superintending Surgeon, Civil Hospital, and Professor of Chemistry, Royal College, Mauritius. Europe, Northern and Western Asia, India, Ceylon and Burmah. Vols. I. and II. New York: D. Appleton & Co. 1892.

A Hand-book of Hygiene and Sanitary Science. By George Wilson, M. A., M. D., F. R. S., Edin., D. P. H., Camb.; Fellow of the Chemical Society; Fellow of the Sanitary Institution of Great Britain; Medical Officer of Health for the Mid-Warwickshire Sanitary District; formerly Medical Officer, H. M. female prison, Woking, and H. M. convict prison, Portsmouth. Seventh edition. Philadelphia: P. Blakiston, Son & Co., 1012 Walnut street. 1892. Price, $5.00.

The Principles and Practice of Bandaging. By Gwilym G. Davis, M. D., Universities of Pennsylvania and Gottingen. Member of the Royal College of Surgeons, England; Assistant Demonstrator of Surgery, University of Pennsylvania; Surgeon of the Out-patient Departments of the Episcopal and Children's Hospital; Assistant Surgeon to the Orthopedic Hospital. Detroit, Mich: George S. Davis. 1891. Price, $3.00.

Transactions of the Texas State Medical Association. Twenty-fourth Annual Session, held at Tyler, Texas, April 26, 27, and 28, 1892. Galveston: J. W. Burson Co., Printers and Publishers. 1892.

A Manual of Obstetrics. By A. F. A. King, M. D., Professor of Obstetrics and Diseases of Women in the Medical Department of the Columbian University, Washington, D. C., and in the University of

Vermont, etc. New (fifth) edition. In one 12mo volume of 450 pages, with 150 illustrations. Cloth, $2.50. Philadelphia: Lea Brothers & Co. 1892.

A Manual of Organic Materia Medica; being a Guide to Materia Medica of the Vegetable and Animal Kingdoms. For the use of Students, Druggists, Pharmacists, and Physicians. By John M. Maisch, Ph. D., Professor of Materia Medica and Botany in the Philadelphia College of Pharmacy. New (fifth) edition, thoroughly revised. In one very handsome 12mo volume of 544 pages, with 270 engravings. Cloth, $3.00. Philadelphia: Lea Brothers & Co. 1892.

A Text-Book of the Principles and Practice of Medicine. For the use of Students and Practitioners. By Henry M. Lyman, M. D., Professor of the Principles and Practice of Medicine in Rush Medical College, Chicago. In one very handsome octavo volume of 926 pages, with 170 illustrations. Cloth, $4.75; leather, $5.75. Philadelphia: Lea Brothers & Co. 1892.

The Essentials of Histology. By Edward A. Schäfer, F. R. S., Jodrell Professor of Physiology in the University College, London. New (third) edition. In one octavo volume of 311 pages, with 325 illustrations. Cloth, $3.00. Philadelphia: Lea Brothers & Co. 1892.

The Student's Handbook of Surgical Operations. By Frederick Treves, F. R. C. S., Surgeon and Lecturer on Anatomy at the London Hospital. In one square 12mo volume of 508 pages, with ninety-four illustrations. Cloth, $2.50. Philadelphia: Lea Brothers & Co. 1892.

Materia Medica and Therapeutics. By L. F. Warner, M. D., Attending Physician St. Bartholomew's Dispensary, New York. Being Vol. V. of the Students' Quiz Series. Pocket size, 244 pages, $1.00. Philadelphia: Lea Brothers & Co. 1892.

An American Text-Book of Surgery for Practitioners and Students. By Charles H. Burnett, M. D., Phineas S. Conner, M. D., Frederic S. Dennis, M. D., William W. Keen, M. D., Charles B. Nancrede, M. D., Roswell Park, M. D., Lewis S. Pilcher, M. D., Nicholas Senn, M. D., Francis J. Shepherd, M. D., Lewis A. Stimson, M. D., William Thomson, M. D., J. Collins Warren, M. D., and J. William White, M. D. Edited by William W. Keen, M. D., LL. D., and J. William White, M. D., Ph. D. Profusely illustrated. Price, $7.00 net, cloth; $8.00, sheep; $9.00, half Russia. Sold by subscription only. Philadelphia: W. B. Saunders, 913 Walnut street. 1892.

Genito-Urinary and Venereal Diseases. By Charles H. Chetwood, M. D., Visiting Surgeon, Demilt Dispensary, Department of Surgery and Genito-Urinary Diseases, New York. Being Volume VIII. of the Students' Quiz Series. Pocket size, 178 pages, $1.00. Philadelphia: Lea Brothers & Co. 1892.

Principles of Theoretical Chemistry, with special reference to the Constitution of Chemical Compounds. By Ira Remsen, M. D., Ph. D., Professor of Chemistry in the Johns Hopkins University, Baltimore. Fourth and thoroughly revised edition. In one handsome royal 12mo volume of 325 pages. Cloth, $2.00. Just ready. Philadelphia: Lea Brothers & Co. 1892.

